# Welcome to aging clinical experimental research!

**DOI:** 10.1007/s40520-024-02716-8

**Published:** 2024-03-04

**Authors:** Nicola Veronese

**Affiliations:** Geriatric Unit, Geriatrics Section, Department of Internal Medicine, University of Palermo, Via del Vespro, 141, 90127 Palermo, Italy

Dear Reader, Dear Friend,

I trust this letter finds you well. As the dawn breaks on a new year, Prof. Stefania Maggi has decided to retire from her position as Editor-in-Chief of *Aging Clinical Experimental Research* (ACER). I wish to express my sincere appreciation for the tireless effort and outstanding contribution of the former editorial board of ACER and, in particular, to Stefania Maggi. Stefania was (and still is!) a great mentor for me. Under her guidance, ACER has further consolidated its position among the most important and well-known journals in the field of Geriatrics. Briefly, ACER was founded in 1989 by Professors Crepaldi, Masoro, and Amaducci. In 2019, ACER formally became the official journal of the European Society for Clinical and Economic Aspects of Osteoporosis, Osteoarthritis and Musculoskeletal Diseases (ESCEO). The Journal Citation Reports published by Clarivate has ranked ACER in the second quartile (Q2) of the category Geriatrics and Gerontology since 2021, when the journal was under the guidance of Prof. Maggi. I sincerely believe that over the past years, the dedication of the editorial board has been instrumental in shaping the journal’s identity and fostering a robust platform for the dissemination of cutting-edge research in geriatric medicine. The commitment to maintaining rigorous standards of publication has not only elevated the journal’s impact, but has also significantly contributed to the advancement of geriatric knowledge and practice. As we bid farewell to some members of the editorial board, I would like to extend my heartfelt gratitude to them for their invaluable contributions, tireless dedication, and unwavering commitment to the pursuit of academic excellence. The collective efforts of the editorial team have left an indelible mark on the journal, establishing it as a reputable source of evidence-based insights for geriatricians, researchers, and healthcare professionals worldwide.

Looking ahead to this New Year, I am honored to take on the role of Editor-in-Chief, and I am excited to introduce and welcome a renewed editorial team that will guide the journal into the next phase of its life. The newly formed editorial board will continue to include esteemed experts in geriatrics and gerontology, facing the new horizons of research dedicated to older people, such as meta-research, epidemiology, and genetics.

In line with the evolving landscape of geriatric medicine, we are committed to introducing fresh perspectives, embracing innovative research methodologies, and fostering interdisciplinary collaboration. Our vision for 2024 and beyond is rooted in a holistic understanding of geriatric care, encompassing not only medical interventions but also psychosocial, ethical, and policy dimensions.

Therefore, I warmly invite all researchers interested in Geriatrics to help us on this exciting journey. Your insights and continued support are invaluable as we strive to maintain the legacy of excellence established by the former editorial board, while ushering in a new era of innovation and inclusivity. In this regard, I would like to remind our readers that some Calls for Papers are still open: in particular, one dedicated to surgery and another to telemedicine, two relevant topics in geriatrics. A final important point is that ACER has become a fully open access (OA) journal as of January 2024. This means that ACER will only be publishing articles as Open Access, making its content available to all readers worldwide, thereby rendering research about older people easier to access and use.

We look forward to receiving your contributions and engaging in fruitful collaborations that will advance the frontiers of geriatric medicine. Together, let us shape ACER into an even more influential and indispensable resource for the geriatric community. Thank you once again for the outstanding work of the previous editorial board and for your ongoing support. May the coming year bring new discoveries, collaborations, and successes to us all.

Nicola.

